# Role of toll-like receptors in human iris pigment epithelial cells and their response to pathogen-associated molecular patterns

**DOI:** 10.1186/1476-9255-11-20

**Published:** 2014-07-16

**Authors:** Kelly Mai, Jeanie JY Chui, Nick Di Girolamo, Peter J McCluskey, Denis Wakefield

**Affiliations:** 1 Inflammation and Infection Research Centre, Department of Pathology, School of Medical Sciences, Faculty of Medicine, University of New South Wales, Sydney, Australia; 2 Department of Ophthalmology, Prince of Wales Hospital, Randwick, NSW, Australia; 3 Save Sight Institute, Sydney Eye Hospital, University of Sydney, Sydney, Australia; 4 UNSW Medicine, University of New South Wales, Sydney, Australia

**Keywords:** TLR, Uveitis, PAMPs, Immunology, IPE

## Abstract

**Background:**

Toll-like receptor (TLR) activation is hypothesized to contribute to inflammatory eye disease including uveitis, yet the distribution pattern of TLRs in human uveal tissues remains poorly described. The purpose of this study was to investigate the expression profile of TLRs in human iris pigment epithelial cells (IPE) at the gene and protein level and examine the effect of pathogen-associated molecular patterns (PAMPs), such as Pam_3_CSK_4_.3HCl, Poly(I:C), lipopolysaccharides (LPS from *E. coli* serotype O111:B4), Flagellin, MALP-2 (macrophage activating lipopeptide-2), Poly(U) and CpGODN2395 on the production of inflammatory mediators including interleukin-8 (IL-8) and monocyte chemotactic protein 1 (MCP-1) from human IPE and retinal pigment epithelial cells (RPE).

**Methods:**

RT-PCR and Western blotting was employed to investigate the expression of TLRs 1–10 in primary IPE and RPE. Secretion of IL-8 or MCP-1 following treatment with PAMPs was measured by ELISA. The role of TLR2, TLR3 and TLR4 in mediating an inflammatory response was investigated using pharmacological TLR inhibitors.

**Results:**

IPE and RPE expressed transcripts for TLR1-6 and 8–10; and proteins for TLR1-6 and 9. IPE secreted IL-8 or MCP-1 in response to Pam_3_CSK_4._3HCl, Poly(I:C), LPS and MALP-2, whereas RPE produced IL-8 only after Poly(I:C), LPS or MALP-2 treatment. TLR inhibitors (OxPAPC, CI-095 and chloroquine) blocked IL-8 secretion in Poly(I:C), LPS or MALP-2-treated IPE and RPE.

**Conclusions:**

Ocular pigment epithelial cells respond to PAMPs through activation of TLRs, particularly TLR2, TLR3 and TLR4. Expression of TLRs in human IPE cells provides a basis for responses to many ocular pathogens and their activation may be involved in the pathogenesis of ocular inflammation.

## Background

Toll-like receptors (TLRs) are a family of phylogenetically conserved transmembrane pattern recognition receptors involved in innate immunity and inflammation. To date, 10 functional TLRs have been reported in humans [1] which recognise unique and highly conserved molecular signatures from microbes known as *pathogen associated molecular patterns* (PAMPs) that include lipopolysaccharides (LPS), flagellin, lipopeptides, lipotechoic acid (LTA), microbial DNA, viral RNAs and others [1].

TLRs have been implicated in ocular inflammation. For example, activation of TLRs by PAMPs due to an initiating mucosal infection and the subsequent immune response has been hypothesised to play a key role in the pathogenesis of anterior uveitis [2]. In addition, expression of TLR2 in human conjunctival epithelial cells was shown to play a significant role in the chronic ocular inflammatory response to *Staphylococcus aureus*[3]. Kezic et al. [4] suggested that both epithelial cells and immune cells play a role in ocular inflammation. Specifically, radiation-resistant, non-bone marrow derived ocular cells, such as iris endothelial cells or nonpigmented ciliary body epithelial cells, play a greater role in the development of endotoxin-induced uveitis (EIU) than bone marrow-derived macrophages and dendritic cells residing in the eye [4]. Therefore, studying TLRs on iris pigment epithelial cells (IPE) and their response to PAMPs may provide an insight into pathogenesis of ocular inflammation, particularly anterior uveitis.

The iris pigment epithelium is the layer of pigmented cells forming the posterior layer of the iris [5]. There is a remarkable resemblance between IPE and retinal pigment epithelial cells (RPE) due to their shared embryonic development [6-8]. *In vitro*, IPE and RPE share functional properties such as phagocytosis and synthesis of cytokines and growth factors [7,9]. Rezai and colleagues showed that IPE elicited phagocytic activity similar to RPE [7]. Non-immune cells, such as IPE and RPE, form an interface between the eye and the environment that is not readily accessible to myeloid cells. By virtue of their ability to detect signals via innate immune receptors, such as toll-like receptors, they are able to recruit myeloid cells, such as neutrophils and macrophages to the site of injury and induce inflammation.

Expression of TLRs has been reported in a number of ocular tissues such as cornea, conjunctiva, sclera and retina [10-14]. Studies have emphasized the importance of the LPS receptor complex (TLR4 and co-receptors CD14 and MD2) expression in ocular tissues and cells such as corneal epithelial cells, cornea stroma fibroblasts, human ciliary body, human iris endothelial cells (TLR4 only), RPE and resident antigen presenting cells in human uvea [14-18]. It has been shown that human RPE express TLRs and are considered to play an important role in posterior ocular inflammation due to their ability to secrete several inflammatory mediators [13,19]. However, little is known about the distribution of TLRs in the uvea, especially the iris. In this study, we investigated the expression pattern and functional significance of TLRs in human ocular pigment epithelial cells (IPE and RPE). This study demonstrated that human IPE and RPE secrete IL-8 and MCP-1 in response to PAMPs, which was partially mediated through TLR activation.

## Methods

### Primary ocular pigment epithelial cells

Donor human eyes were obtained from Lions NSW Eye Bank, Sydney, Australia within 16 hours of death. Collection of all human samples followed the Tenants of the Declarations of Helsinki and had institutional Human Research Ethics Committee approval (HERC11190; HERC10026). Donor matched IPE and RPE were isolated from human eyes (n = 3 donors) using a previously described method [20] with minor modifications. Briefly, globes were bisected into anterior and posterior segments and the retina was peeled away to expose the RPE. The iris was removed and placed into a culture dish. Ocular tissues were incubated in 0.25% Trypsin/EDTA at 37°C for 3 hours and IPE and RPE were mechanically removed. Isolated cells were washed in phosphate buffered saline (PBS) and collected by centrifugation. Cell suspensions were transferred to T-25 culture flasks containing epithelial cell medium (ScienCell Research Laboratories, San Diego, CA) supplemented with 2% FBS, 100 U/ml penicillin and 100 μg/ml streptomycin and maintained at 37°C in an atmosphere of 5% CO_2_. Primary IPE (≤passage 4) and RPE (≤passage 5) were used in all experiments. Cells were probed with an anti-human pan-cytokeratin antibody (Clone C-11, Sigma-Aldrich, St Louis, MO) to ensure they were of epithelial origin before experimentation (see Additional file 1). The spontaneously arising retinal pigment epithelial cell line (ARPE-19), previously characterised by Dunn et al. [21], were cultured in a 1:1 mixture of DMEM and Ham’s F12 medium supplemented with 2% FBS, 100 U/ml penicillin and 100 μg/ml streptomycin. Human acute monocytic leukemia cells (THP-1) were cultured in RPMI 1640 medium supplemented with 10% FBS, 100 U/ml penicillin and 100 μg/ml streptomycin as described previously [22] and both were used as positive controls.

### Antibodies and TLR ligands

Anti-TLR, −GAPDH and isotype control antibodies were purchased from eBioscience (San Diego, CA), Imgenex (San Diego, CA) and R&D systems (Minneapolis, MN; see Table 1 for details). Secondary antibodies biotinylated anti-goat, mouse or rabbit immunoglobulins and HRP-conjugated streptavidin were purchased from DAKO (Glostrup, Denmark). TLR ligands used for functional assays were either endotoxin-free or endotoxin-minimized (see Table 2 for details).

**Table 1:** Antibodies used for Western blotting

**Antigen specificity**	**Antibody subtype**	**Label**	**Clone**	**Manufacturer**	**Final concentration (μg/ml) or dilution**
TLR1	Goat IgG	biotin	-	R&D systems	0.2
TLR2	Goat IgG	biotin	-	R&D systems	0.2
TLR3	Mouse IgG_1,_ k	-	40C1285.6	Imgenex	2
TLR4	Goat IgG	biotin	-	R&D systems	0.2
TLR5	Mouse IgG_2a,_ k	-	19D759.2	Imgenex	2
TLR6	Rabbit IgG	-	-	Imgenex	2
TLR7	Rabbit IgG	-	-	Imgenex	2
TLR8	Mouse IgG_1,_ k	-	44C143	Imgenex	2
TLR9	Mouse IgG_1,_ k	-	26C593.2	Imgenex	2
TLR10	Mouse IgG_1_	-	158C1114	Imgenex	2
Isotype	Mouse IgG_2a,_ k	-	-	eBioscience	10
GAPDH	Mouse IgG_1_	-	GAPDH 1D4	Imgenex	1:1000 dilution

**Table 2:** TLR ligands set for functional assays

**Ligands or agonists**	**Product description**	**Endotoxin content**	**Toll-like Receptors**	**Manufacturer**
Pam_3_CSK_4._3HCl	Synthetic tripalmitoylated lipopeptide, an analog of the immunologically active N-terminal portion of bacterial lipoprotein	< 0.01 EU/μg	TLR1/2	TLR Ligands Set I (Apotech®, Switzerland)
Poly(I:C)	Synthetic mimetic of viral double-stranded RNA	< 0.01 EU/μg	TLR3	
Flagellin	Isolated from *Salmonella typhimurium* strain 14028; highly conserved molecules among gram negative and gram positive bacteria, especially in 170 N-terminal and 100 C-terminal amino acid	< 0.1 EU/μg	TLR5	
MALP-2	Synthetic macrophage-activating lipopetides-2 isolated from *Mycoplasma fermentans*	< 0.01 EU/μg	TLR2/6	
Poly(U)	Synthetic analog of single-stranded RNA	< 0.1 EU/μg	TLR7/8	
CpG ODN2395	Synthetic oligodeoxynucleotides containing unmethylated deoxycytosine-deoxyguanosine (CpG) motif, mimicking the effects of bacterial DNA	< 0.1 EU/μg	TLR9	
LPS (O111:B4)	Extracted from *Escherichia coli* serotype O111:B4 and purified by ion exchange	-	TLR4	Sigma-Aldrich, St. Louis, MO

### Real-time and reverse transcription polymerase chain reaction (RT-PCR)

Total RNA was isolated from IPE and RPE using TRI reagent® (Sigma-Aldrich, USA) according to manufacturer’s instruction. Total RNA (1 μg) was reverse transcribed into cDNA using SuperScript™ III RT system (Invitrogen, Grand Island, NY) with oligo dT primers. Each PCR reaction mixture contained 1 μl of cDNA template, 2 μl of TLRs primer mix (Table 3), 10 μl of LightCycler® 480 Sybr green I master (Roche Diagnostics, IN, USA) and RNase-free water in a total volume of 20 μl. The assay was performed in a LightCycler® 480 Real-Time PCR instrument (Roche) with an initial activation at 95°C for 10 minutes, followed by amplification of 45 cycles with 2 steps (denaturation at 95°C for 10 seconds, combined annealing and extension at 60°C for 30 seconds). The experiment was performed in duplicate and included negative controls which contained no cDNA template. The concentration of TLR genes was determined using the comparative threshold cycle (CT) number and normalized to that of GAPDH.

**Table 3:** TLR specific primers and RT-PCR conditions

**Genes**	**Sequence (5′ to 3′ direction)**	**Annealing temperature (°C)**	**Cycles**	**Amplicon size (bp)**
TLR1	F: AAAAGAAGACCCTGAGGGCC	62	35	340
	R: TCTGAAGTCCAGCTGACCCT			
TLR2	F: GTACCTGTGGGGCTCATTGT	62	35	191
	R: CTGCCCTTGCAGATACCATT			
TLR3	F: AAATTGGGCAAGAACTCACAGG	60	35	320
	R: GTGTTTCCAGAGCCGTGCTAA			
TLR4	F: TACAAAATCCCCGACAACCTC	60	35	264
	R: AGCCACCAGCTTCTGTAAACT			
TLR5	F: TGCATTAAGGGGACTAAGCCTC	60	35	351
	R: AAAAGGGAGAACTTTAGGGACT			
TLR6	F: TCTTGGGATTGAGTGCTATGA	60	35	337
	R: GTCGTTTCTATGTGGTTGAGG			
TLR7	F: TCCAGTGTCTAAAGAACCTGG	60	38	352
	R: TGGTAAATATACCACACATCCC			
TLR8	F: TAATAGGCTGAAGCACATCCC	60	35	621
	R: TCCCAGTAAAACAAATGGTGAG			
TLR9	F: GTGCCCCACTTCTCCATG	60	35	260
	R: GGCACAGTCATGATGTTGTTG			
TLR10	F: TGACCACAATTCATTTGACTACTC	60	35	478
	R:TTGAATACTTTTGGGCAAGCACC			
GAPDH	F:ACCACAGTCCATGCCATCAC	60	28	452
	R:TCCACCACCCTGTTGCTGTA			
Genes	Sequence	Accession number	Cycles	Amplicon size (bp)
TLR2*	Sequences refer to Qiagen	NNM_003264	45	92
TLR3*		NNM_003265	45	90
TLR4*		NNM_138554	45	111
TLR6*		NNM_006068	45	78
GAPDH*		NNM_001256799	45	95

F = forward primer; R = reverse primer; bp = base pair. *Primer mix used for real-time PCR.

For reverse transcription PCR, each reaction mixture contained 1 μl of cDNA, 200 nM each of TLR forward and reverse primer (Table 3), 200 μM dNTPs, 2.5 mM MgCl, 1 U Platinum *Taq* DNA polymerase (Invitrogen) and made up to 20 μl with DEPC-treated water. PCR was performed using a GeneAmp® PCR system 9700 (Applied Biosystems, Foster City, CA) with the following conditions: initial denaturation at 95°C for 2 minutes, followed by 35 cycles with 3 steps (denaturation at 95°C for 30 seconds, annealing at 60-62°C, depending on GC and AT contents of primers, for 30 seconds, and extension at 72°C for 30 seconds) and a final extension at 72°C for 2 minutes. PCR products were displayed on 2% agarose gels after electrophoresis. Images were taken using a Molecular Imager® Gel Doc™ system (Bio-Rad, Hercules, CA) after staining with GelRed Nucleic acid stain (Biotium Inc, Hayward, CA). TLR transcripts were quantified by densitometry and normalised against GAPDH which served as the loading control.

### Western blotting

Whole cell lysates from IPE and RPE were prepared as previously described [23]. Briefly, cells were incubated for 30 minutes in ice cold lysis buffer (0.1% SDS, 0.5% NP-40 in 50 mM Tris–HCl pH 7.4) supplemented with a protease inhibitor cocktail (Complete Protease Inhibitor Cocktail, Roche, Mannheim, Germany). Lysates were centrifuged at 10,000 g for 10 minutes at 4°C and protein concentration measured using the DC Protein Assay (Bio-Rad, Hercules, CA). Proteins from lysates (20 μg) were separated by 10% SDS-PAGE under non-reduced conditions, transferred to PVDF membranes, then blocked in 5% skim milk/Tris buffered saline (TBS) at 4°C for 16 hours. Membranes were subsequently incubated with appropriate primary antibodies (Table 1) in 1% BSA/TBS for 1 hour at room temperature, followed by one hour incubation in biotinlyated anti-goat, -mouse or -rabbit IgG (1:2000 dilution; Dako) and HRP-conjugated streptavidin (1:1000 dilution; Dako). Membranes were developed using Western Lightning™ Plus-ECL Enhanced Chemiluminescence Substrate (PerkinElmer Inc, Waltham, MA).

### *In vitro* cell culture studies

Primary IPE and RPE were seeded at a density of 3 × 10^3^ cells/ml in 24-well plates (NUNC, Denmark) and used for experimentation once they reached confluence. Briefly, cells were washed extensively in sterile PBS to remove residual serum, followed by serum starvation for 16 hours. On the following day, cells were stimulated with various concentrations of TLR ligands (Table 2) under serum free conditions at 37°C for 24 hours. Conditioned media was collected and centrifuged at 10,000 g for 10 minutes to eliminate cell debris. IL-8 and MCP-1 content was measured using Human CXCL8/IL-8 and CCL2/MCP-1 DuoSet ELISA Development kit (R&D systems, Minneapolis, MN), respectively.

For TLR inhibition studies, cells were cultured to confluence in 24-well plates and serum starved as described above. On the following day, they were incubated with various concentrations of OxPAPC (TLR2 and TLR4 inhibitor, InvivoGen, San Diego, CA) [24] for 30 minutes, or CI-095 (TLR4 inhibitor, InvivoGen, San Diego, CA) [25,26] for 60 minutes at 37°C, or chloroquine (TLR3 inhibitor, InvivoGen, San Diego, CA) [27] for 60 minutes, then stimulated with LPS from *E. coli* serotype O111:B4 (Sigma-Aldrich, St. Louis, MO), MALP-2 (Apotech®, Switzerland) or Poly(I:C) (Apotech®, Switzerland) for 24 hours. Conditioned media was collected and the IL-8 or MCP-1 levels measured by ELISA as described above. Trypan blue exclusion was used to assess cell viability in the presence of high concentrations of the inhibitors (0.1 - 10 μg/ml for both OxPAPC and CI-095; 10–50 μM for chloroquine) in order to monitor the toxicity of the inhibitors to the cells.

### Statistical analysis

Experiments were performed in triplicate and values were presented as mean ± standard deviation. One-way ANOVA followed by Dunnett’s post test or Bonferroni’s multiple comparison test were used to compare responses between controls and treatment groups; a *p* value of <0.05 was considered significant. All data analysis was performed using GraphPad Prism (version 5, GraphPad Software).

## Results

### Human IPE and RPE express TLRs

**Figure 1:**
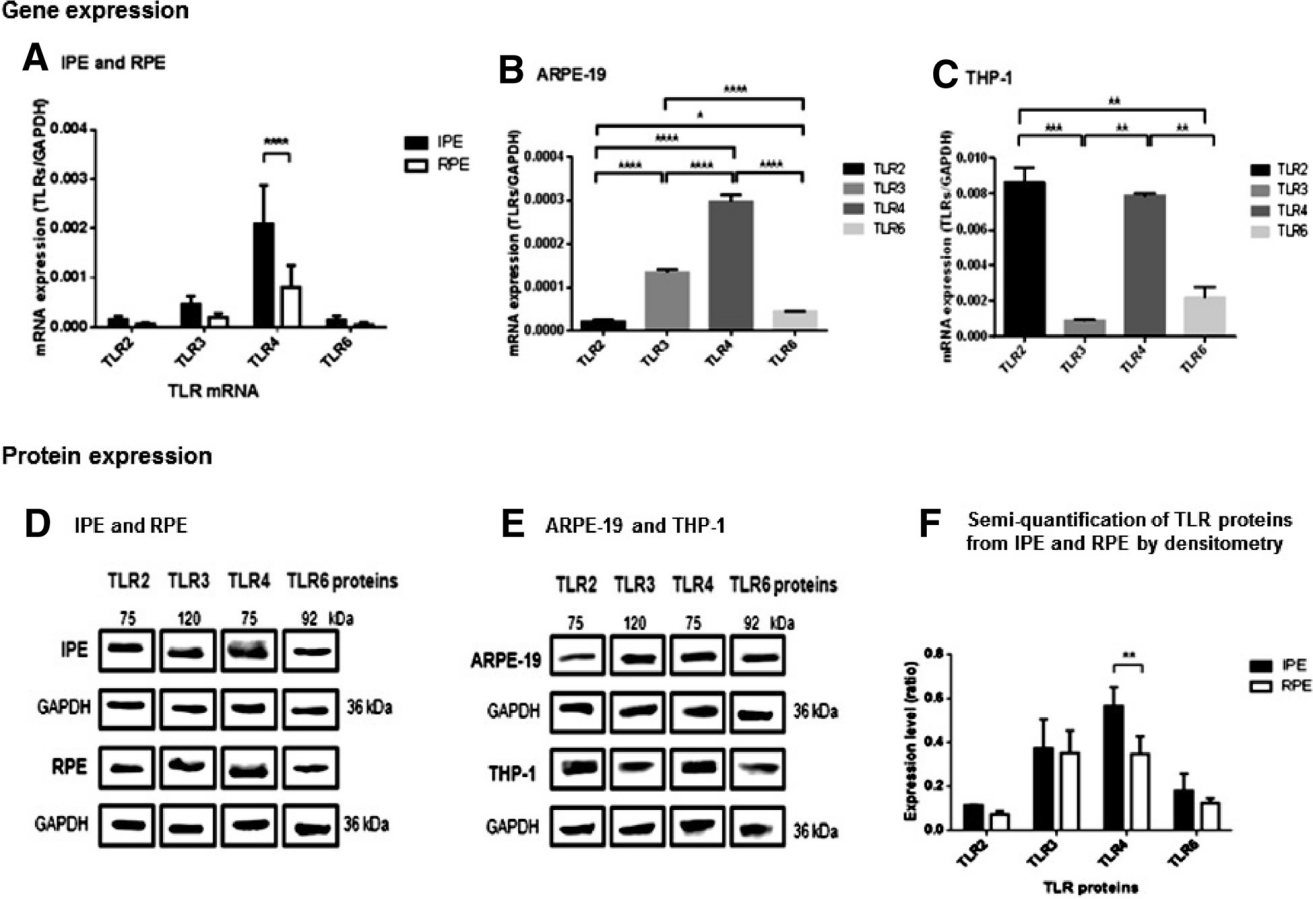
**Expression of TLR2, −3, −4 and −6 mRNA and proteins in human IPE and RPE.** Human IPE and RPE **(A)** from the same donor were cultured to confluence and expression of TLR2, −3, −4 and −6 mRNA and proteins was investigated by real-time PCR **(A, B and C)** and Western blotting **(D, E and F)** using specific primers and antibodies, respectively. The concentration of genes was measured using the comparative threshold cycle number and normalised to that of the GAPDH with ARPE-19 **(B)** and THP-1 **(C)** served as positive controls. Data represents mean ± SD (N = 3). Two-way ANOVA and Bonferroni’s multiple comparison test was used to analyse the data for A, **p < 0.01, ****p < 0.0001. One-way ANOVA and Turkey’s multiple comparison test was used to analyse the data for B and C, *p < 0.05, **p < 0.01, ***p < 0.001, ****p < 0.0001. Results are representative of three independent experiments (the results are consistent from three donors).

Real time-PCR analysis revealed that human IPE and RPE expressed mRNA for TLR2, −3, −4 and −6 (Figure 1A) which were also expressed in positive controls ARPE-19 and THP-1 cell lines (Figure 1B and C). Negative control reactions containing no cDNA template or no RT enzyme generated no amplicons (Data not shown). Interestingly, expression of TLR4 mRNA was higher than that of the other genes (TLR2, −3 and −6) in IPE, RPE and ARPE-19 whereas both TLR2 and −4 genes were highly expressed in THP-1. For example, IPE expressed higher TLR4 than TLR2 genes by 20.8 fold, (Table 4) and a similar pattern was observed in RPE and ARPE-19. Western blotting was performed to further confirm the expression of TLR proteins in human IPE and RPE. Specific bands representing TLR2, −3, −4 and −6 proteins were detected from IPE and RPE (Figure 1D and F) as well as in ARPE-19 and THP-1 cell lines (Figure 1E) and the results were consistent. An additional data presented in Appendix (see Additional file 2) showed gene and protein expression of other TLRs.

**Table 4:** Fold changes in TLR2, −3, −4 and −6 mRNA expression in IPE, RPE, ARPE-19 and THP-1 cells

	**IPE**	**RPE**	**ARPE-19**	**THP-1**
Genes	Fold changes			
***TLR2***	1	1	1	1
***TLR3***	4.21	2.95	6.86	0.12
***TLR4***	20.82	16.64	15.3	0.92
***TLR6***	1.22	1.14	2.31	0.25

Results were normalised to GAPDH and presented as fold changes compared to *TLR2* gene.

For example, ARPE-19 cells expressed higher *TLR3* than *TLR2* genes by 6.86 folds.

### IPE and RPE secrete IL-8 and MCP-1 in response to PAMPs

**Figure 2:**
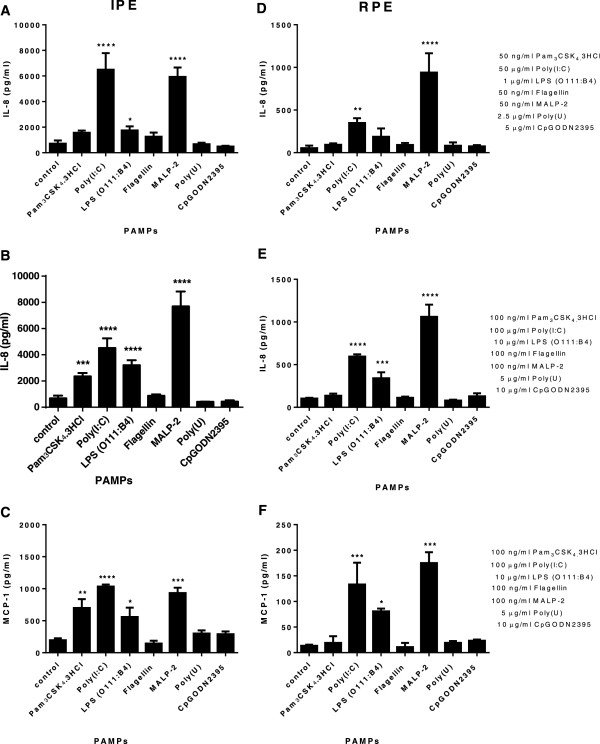
**Human IPE and RPE exhibit different sensitivities in response to PAMPs.** IPE **(A, B and C)** and RPE **(D, E and F)** from the same donor were stimulated with PAMPs such as Pam_3_CSK_4_.3HCl, Poly(I:C), LPS (serotype O111:B4), Flagellin, MALP-2 (macrophage activating lipopeptide-2), Poly(U) and CpGODN2395 at a lower **(A and D)** and higher dose **(B, C, E and F)**, under serum free condition for 24 hours (control = basal media). Conditioned media were collected and assayed for IL-8 or MCP-1 by ELISA. Data represents mean ± SD (N = 3). One-way ANOVA and Dunnett’s posttest was used to compare ligand-treated samples to controls, *p < 0.05, **p < 0.01, ***p < 0.001, ****p < 0.0001. Results are representative of three experiments (the results are consistent from three donors).

**Figure 3:**
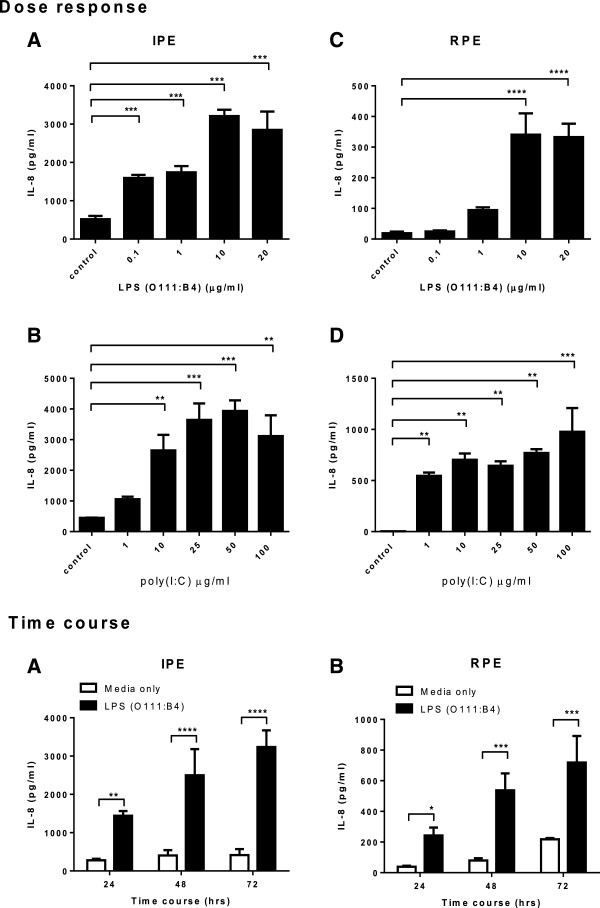
**Dose response and time course of IL-8 secretion from LPS or Poly(I:C) treated IPE and RPE.** Panel 1: Dose response; IPE **(A and B)** and RPE **(C and D)** from the same donor were stimulated with various concentrations of LPS (serotype O111:B4) or Poly(I:C) under serum free condition for 24 hours (control = basal media). Panel 2: Time course; LPS stimulation was performed at a maximal dose of 10 μg/ml under serum free condition for 24, 48 and 72 hours (□ = media only; ■ = LPS stimulation). Conditioned media were collected and assayed for IL-8 by ELISA. Data represents mean ± SD (N = 3). One-way ANOVA and Dunnett’s post test was used to compare LPS or Poly(I:C) treated samples to controls from panel 1, **p < 0.01, ***p < 0.001, ****p < 0.0001. Two-way ANOVA and Bonferroni’s multiple comparison test were used to analyse the data from panel 2. *p < 0.05, **p < 0.01, ***p < 0.001, ****p < 0.0001. Results are representative of three experiments.

IPE secreted significantly increased levels of IL-8 in response to Poly(I:C), LPS and MALP-2 (macrophage-activating lipopeptide-2) (Figure 2A). A similar profile was observed when IPE was stimulated with higher (2x) dose of the same ligands as well as Pam_3_CSK_4_.3HCl (Figure 2B). Increasing concentration of Poly(I:C) beyond 100 μg/ml did not enhance IL-8 production suggesting that a maximal IL-8 secretion was observed at a dose of 50 μg/ml. However, flagellin, poly(U) and CpG ODN2395 (synthetic oligodeoxynucleotides) did not enhance IL-8 secretion (Figure 2A, B). TLR7 and 8 were not expressed by IPE or RPE (see Additional file 2) which may explain why they do not respond to Poly(U) stimulation. Whereas RPE secreted IL-8 in response to Poly(I:C) and MALP-2 but did not respond to Pam_3_CSK_4_.3HCl, LPS (O111:B4), flagellin, poly(U) or CpG ODN2395 (Figure 2D). A similar response profile, except LPS stimulation, was observed when RPE were stimulated with higher (2x) doses of the same ligands (Figure 2E). A similar trend was seen when MCP-1 was assayed. Both IPE and RPE secreted MCP-1 in response to the same ligand stimulation (Figure 2C, F), which is consistent with the IL-8 production profile. Interestingly, IPE were more sensitive than RPE in their response to PAMPs as more IL-8 and MCP-1 was produced by IPE than RPE at the same dose of ligand stimulation. LPS and Poly(I:C) dose–response experiments were performed and maximal IL-8 secretion was observed at a final concentration of 10 μg/ml for both cell types (Figure 3 panel 1, A, C) and 50 μg/ml for IPE (Figure 3 panel 1, B), respectively. However, maximal IL-8 secretion was not seen from RPE (Figure 3 panel 1, D) at the concentrations tested here. Higher dose of Poly(I:C) stimulation (>100 μg/ml) is required to investigate maximal IL-8 secretion for RPE. Time course experiments were performed at 24, 48, 72 and 96 hours. Secretion of IL-8 from IPE and RPE in response to LPS (O111:B4) was observed at 24, 48 and 72 hours (Figure 3 panel 2, A, B). It was noted that accumulated effect of IL-8 occurred at later time points (72 and 96 hours; i.e. IL-8 level was increased due to cultured medium was vaporized beyond 72 hours) therefore subsequent experiments were performed at 24 hours.

### TLR inhibitors block IL-8 secretion in IPE and RPE stimulated cells

**Figure 4:**
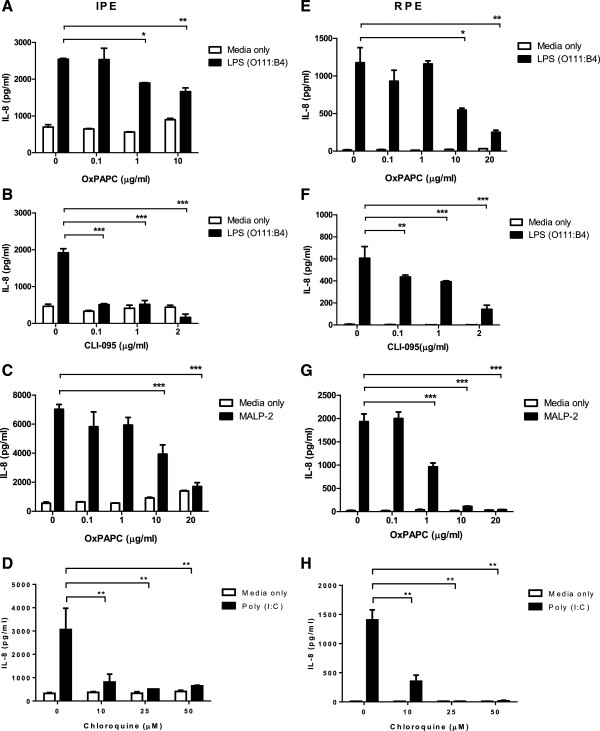
**TLR inhibitors suppress IL-8 secretion from LPS, MALP-2 or Poly(I:C) treated IPE and RPE.** IPE **(A-D)** and RPE **(E-H)** from the same donor were stimulated with LPS (serotype O111:B4; 10 μg/ml - maximal dose), MALP-2 (100 ng/ml) or Poly(I:C) (10 μg/ml) in the presence of OxPAPC (TLR2 and 4 inhibitors), CI-095 (TLR4 inhibitor) and chloroquine (TLR3 inhibitor) respectively, at various concentrations (□ = media only; ■ = LPS stimulation). Conditioned media were collected and assayed for IL-8 by ELISA. Data represents mean ± SD (N = 3). One-way ANOVA and Bonferroni’s multiple comparison test were used to analyse the data. IL-8 secretion by LPS, MALP-2 or Poly(I:C) treated IPE and RPE was significantly inhibited in the presence of OxPAPC, CI-095 or chloroquine in comparison to exposed to the ligand alone, *p < 0.05, **p < 0.01, ***p < 0.001. Results are representative of three experiments (two studies from the same donor and the results are consistent from three donors).

OxPAPC (TLR2 and 4 inhibitor), CI-095 (TLR4 inhibitor), chloroquine (TLR3 inhibitor) significantly inhibited IL-8 secretion in both IPE and RPE challenged with LPS (Figure 4A, B, E and F), MALP-2 (Figure 4C and G) or Poly(I:C) (Figure 4D and H), confirming their specificity to TLR2, −3 and −4 respectively. Negative controls containing various concentrations of inhibitors were also included to monitor the level of IL-8 secretion with no ligand stimulation (Figure 4). The negative controls contained minimal level of IL-8, similar to the samples containing basal medium alone. In addition, ~90% of IPE and RPE remained viable in the presence of 0.1 - 10 μg/ml for both OxPAPC and CI-095; 10–50 μM for chloroquine (see Additional file 3), suggesting that cell death did not account for reduction in IL-8 secretion (i.e. the inhibitors were not toxic to the cells). Therefore, IPE and RPE secreted IL-8 in response to LPS and MALP-2 stimulation is likely mediated through TLR4 and TLR2/6, respectively, whereas Poly(I:C) is through TLR3 signalling.

## Discussion

This is the first report to characterise the expression profile of TLRs in human IPE. IPE and RPE expressed TLR1-6 and 8–10 transcripts; TLR1-6 and 9 proteins (see Additional file 2) however TLR7, 8 and 10 proteins were not detected. Real-time PCR was performed to further confirm whether there is differential expression of TLR2, −3, −4 and −6 genes in both cell types (Figure 1). Additionally, IPE and RPE expressed higher TLR4 levels than TLR2 and this may account for its higher responsiveness to LPS than Pam_3_CSK_4_.3HCl stimulations (Figures 1 and 2). IPE may have been more responsive to LPS stimulation than RPE due to the fact that they expressed higher TLR4 levels than RPE (Figures 1 and 2). The level of TLR3 mRNA and proteins was not significantly different between the two cell types under basal medium (Figure 1 A). However, IPE and RPE showed different sensitivity in response to Poly(I:C) stimulation. In addition, both cells showed higher expression of TLR4 mRNA and proteins than that of TLR3. They secreted higher level of IL-8 in response to Poly(I:C), compared to LPS stimulation (Figure 2). It is noteworthy that the experiments conducted for Figure 1 were performed with basal medium. Hence, further study is needed to investigate the level of TLR2, −3, −4, −6 mRNA and proteins following ligand stimulation. Nevertheless the presence of TLR proteins detected by Western blotting may not necessarily reflect on the amount of the proteins involved in the signalling. Some of the proteins may be degraded or undergo post-translational modification for functioning. Interestingly, TLR7 mRNA was not detected in either cell type and this may well explain the absence of TLR7 proteins. The lack of TLR8 and 10 proteins in ocular pigment epithelial cells may reflect the low expression levels, which in turn may influence their response to PAMPs.

IPE express TLR4 *in vitro* consistent with our earlier findings that these cells express a functional LPS receptor complex (TLR4, MD-2, and CD14) *in vitro* and secreted several pro-inflammatory cytokines including IL-6, IL-8, MCP-1, IP-10, MIP-1-beta and RANTES in response to LPS stimulation [23]. Here, we showed that RPE expressed all TLR transcripts except TLR7. In contrast, Kumar and colleagues reported that human RPE express all TLR mRNAs except TLR8 [13]. This difference may be due to donor variation, different culture conditions or different time in culture (passage 2–5 in our study versus passage 7–12). In our studies, both IPE and RPE became less responsive to TLR ligands with increasing passage and failed to respond beyond generation 5 and 6 respectively (results not shown). Our results seem more reliable as low passage number cultures and donor-matched primary IPE and RPE were used, and the medium was formulated to promote optimal epithelial growth.

Previous studies used a well-characterised cell line, ARPE-19 (spontaneous arising retinal pigment epithelial cells), as representative of retinal pigment epithelial cells (RPE). In our previous study, we showed that there is a differential expression of CD14, a co-receptor of TLR4, between IPE and ARPE-19 [23]. However, expression of CD14 from donor-matched primary IPE and RPE was not significantly different between the two cell types (data not shown). In addition, it has been shown that an elevated expression of proteins associated with microtubule cytoskeleton and IL-18 production was observed in ARPE-19 in comparison to RPE [28,29]. Therefore, the results generated from ARPE-19 should be interpreted with caution, as they may not be a reliable substitute for the primary cultured cells.

Although IPE and RPE possess a similar TLR expression profile, IPE appears to be more sensitive to PAMPs than RPE in culture and this observation was consistent across all three IPE and RPE matched donors (Figure 2). Culture supernatants confirmed that both IPE and RPE secreted a similar amount of total protein. IPE secreted maximal levels of IL-8 at 50 μg/ml following Poly(I:C) stimulation, whereas RPE required significantly higher dose of poly(I:C) (>100 μg/ml) for maximal IL-8 secretion. It was noted that high dose of LPS stimulation (10 μg/ml) was needed for a significant response in RPE. A recent finding suggests that LPS can be recognised in a TLR4-independent, caspase 11-dependent manner in mice and this activation was serotype specific, occurring in response only to *E. coli* serotype O111:B4 [30]. In our study the same serotype of LPS from *E. coli* was used. Therefore it cannot be ruled out the possibility that LPS mediated IL-8 secretion in RPE may be TLR4-independent.

The pro-inflammatory cytokines, IL-8, was chosen for this study based on our previous findings: (i) that IL-8, MCP-1, IP-10, RANTES and MIP-1*b* were significantly increased in aqueous humour of patients with active stage of anterior uveitis and this correlated with the clinical severity of the disease [31]; (ii) IL-8 has been shown to contribute to the chemotactic signal for the recruitment of leukocytes in EIU [32]. Therefore, IL-8 is an important mediator of the inflammatory response in clinical settings and in experimental animal models of uveitis.

MCP-1 is one of the key chemokines that regulate migration and infiltration of monocytes\macrophages to the sites of inflammation due to infection or tissue injury. The fact that IPE and RPE secreted both IL-8 (neutrophil chemoattractant) and MCP-1 in response to PAMPs are consistent with their role in innate immune responses and inflammation. IPE and RPE are regarded as “guardians” of the eye as they sense danger signals and consequent initiation of an inflammatory response. Whether the differences in their responsiveness to PAMPs is due to the nature of the cells; differential expression of TLRs and/or their co-receptors or different down-stream TLR signalling pathways or mRNA and protein stability, remains to be ascertained. Differential expression of TLRs and their co-receptors in ocular pigment epithelial cells may influence their response to PAMPs.

The role of TLR2, −3 and −4 in mediating cytokine response in Poly(I:C), LPS or MALP-2-treated IPE and RPE was also investigated using TLR inhibitors (OxPAPC, CI-095 and chloroquine; Figure 4). OxPAPC is an inhibitor of TLR2 and -4 signaling by competing with CD14, LBP and MD2, the accessory molecules that interact with bacterial lipids [24]. Interestingly, IPE and RPE showed different sensitivity to OxPAPC. For example, IPE were more sensitive to OxPAPC in suppressing LPS mediated IL-8 secretion than were RPE cells (Figure 4A and E), but less sensitive to suppress MALP-2 mediated IL-8 secretion than RPE (Figure 4C and G). The difference could be due to different levels of accessory molecules of TLR4, such as CD14, LBP and MD2 on cell surface between the two cell types. CI-095 (also known as TAK-242) suppresses TLR4 signaling via its action on the intracellular domain of TLR4 and inhibits the production of nitric oxide and pro-inflammatory cytokines [25,26]. Chloroquine is a lysosomotropic agent that prevents endosomal acidification thus blocks signalling of intracellular TLRs [27]. Both IPE and RPE showed similar response/sensitivity to CI-095 and chloroquine, which are potent inhibitors for TLR4 and TLR3, respectively. The fact that OxPAPC, CI-095 and chloroquine inhibited IL-8 secretion from LPS, MALP-2 or Poly(I:C)-treated IPE and RPE, suggests a role for TLR4 and possibly TLR2, TLR3 in LPS, MALP-2, Poly(I:C) mediated inflammatory responses.

A limitation of our study is that we employed a cell culture system. Nevertheless, animal studies support the concept that TLRs play a role in the pathogenesis of experimental autoimmune uveitis and EIU [4,33-35]. In addition, animal studies also provide an insight into physiological relevance of pro-inflammatory cytokine production *in vivo*. For example, Allensworth et al. showed the potential of TLRs to trigger uveitis in mice [33]. They concluded that all TLR agonists tested induced inflammation in the mouse eye with a marked increase of TNF-α, IL-6, IP-10, MCP-1 and KC and relatively little production of IFN-γ, IL-10, IL-12, IL-17, IL-1β, IL-4 or RANTES. In the current study, we have shown for the first time that cultured IPE cells express functional TLRs and respond to PAMPs through activation of TLRs, particularly TLR2, TLR3 and TLR4. This study extends the current knowledge of the role of TLR activation in iris culture and uveal innate immune mechanism in the pathogenesis of ocular inflammation.

## Conclusion

Our results demonstrate that IPE cells express functional TLRs and their activation may have implications for the pathogenesis of ocular inflammation.

## Abbreviations

IPE: Iris pigment epithelial cells; RPE: Retinal pigment epithelial cells; PAMPs: Pathogen-associated molecular patterns; TLRs: Toll-like receptors; LPS: Lipopolysaccharides; MALP-2: Macrophage activating lipopeptide-2.

## Competing interests

Authors declare no conflict of interest.

## Authors' contributions

KM designed, performed and analysed all the experiments and prepared the manuscript. JJYC assisted in data analyses, presentation and helped to draft the manuscript. NDG, PJM and DW designed experiments, supervised all aspects of the project and revised the manuscript. All authors read and approved the final manuscript.

## Supplementary Material

Additional file 1 **Human IPE and RPE stained for cytokeratin.** The primary IPE and RPE, cultured from iris and retina, of human donors, were probed with anti-pan cytokeratin antibody (1:200 dilution; **A**) and an isotype control antibody (mouse IgG_1_; 1: 200 dilution). Immunohistochemistry was performed to confirm the epithelial origin of primary IPE and RPE. A representative of cytokeratin stained IPE was shown in **A** and no staining was seen in the negative control (IgG_1_). The result was confirmed by flow cytometry **(B)**. The peak representing cytokeratin stained cells (in pink) was shifted away from that of the isotype control antibody (in grey). Click here for file

Additional file 2 **Expression of TLR transcripts and proteins in human IPE and RPE.** Human IPE (lane 1) and RPE (lane 2) from the same donor were cultured to confluence and expression of TLR1 to TLR10 genes and proteins was investigated by reverse transcription PCR **(A)** and Western blotting **(B)** using specific human TLR1 to TLR10 primers and antibodies, respectively. M= 100 bp DNA ladder (100, 200, 300, 400, 500, up to 1000 bp from bottom to top). TLR mRNA expression was measured by densitometry and normalised against GAPDH which served as a loading control. Normalised TLR mRNA expression levels are presented as mean ± SD (N=3) **(C)**. Two-way ANOVA and Bonferroni’s multiple comparison test were used to analyse the data, *p< 0.05, **p< 0.01. Expression of TLR7 mRNA (A); TLR8 and TLR10 proteins (B) were not detected in both IPE and RPE. Results are representative of three experiments. Click here for file

Additional file 3 **Viability of IPE and RPE in the presence of OxPAPC, CI-095 and chloroquine.** IPE and RPE were cultured in various concentrations of OxPAPC **(A and D)**, CI-095 **(B and E)** and chloroquine **(C and F)** for 24 hours. The cells were subsequently detached from culture plates by trypsin, followed by assessment of viability using Trypan blue. Data represents mean ± SD (N=3). One-way ANOVA and Dunnett’s post test was used to compare inhibitor-treated samples to controls. Both IPE and RPE remained ~90% viable in the presence of the high concentrations of TLR inhibitors. There was no difference in cell viability between control and inhibitor-treated cells. Click here for file
