# Supplementary material for: Role of toll-like receptors in human iris pigment epithelial cells and their response to pathogen-associated molecular patterns
Source: J Inflamm (Lond). 2014 Jul 16;11:20. doi: 10.1186/1476-9255-11-20 (PMC4118659; doi:10.1186/1476-9255-11-20)

Additional data

File 1 Human IPE and RPE stained for cytokeratin. The primary IPE and RPE, cultured from iris and retina, of human donors, were probed with anti-pan cytokeratin antibody (1:200 dilution; **A**) and an isotype control antibody (mouse IgG_1; 1: 200 dilution). Immunohistochemistry was performed to confirm the epithelial origin of primary IPE and RPE. A representative of cytokeratin stained IPE was shown in **A** and no staining was seen in the negative control (IgG). The result was confirmed by flow cytometry (**B**). The peak representing cytokeratin stained cells (in pink) was shifted away from that of the isotype control antibody (in grey).

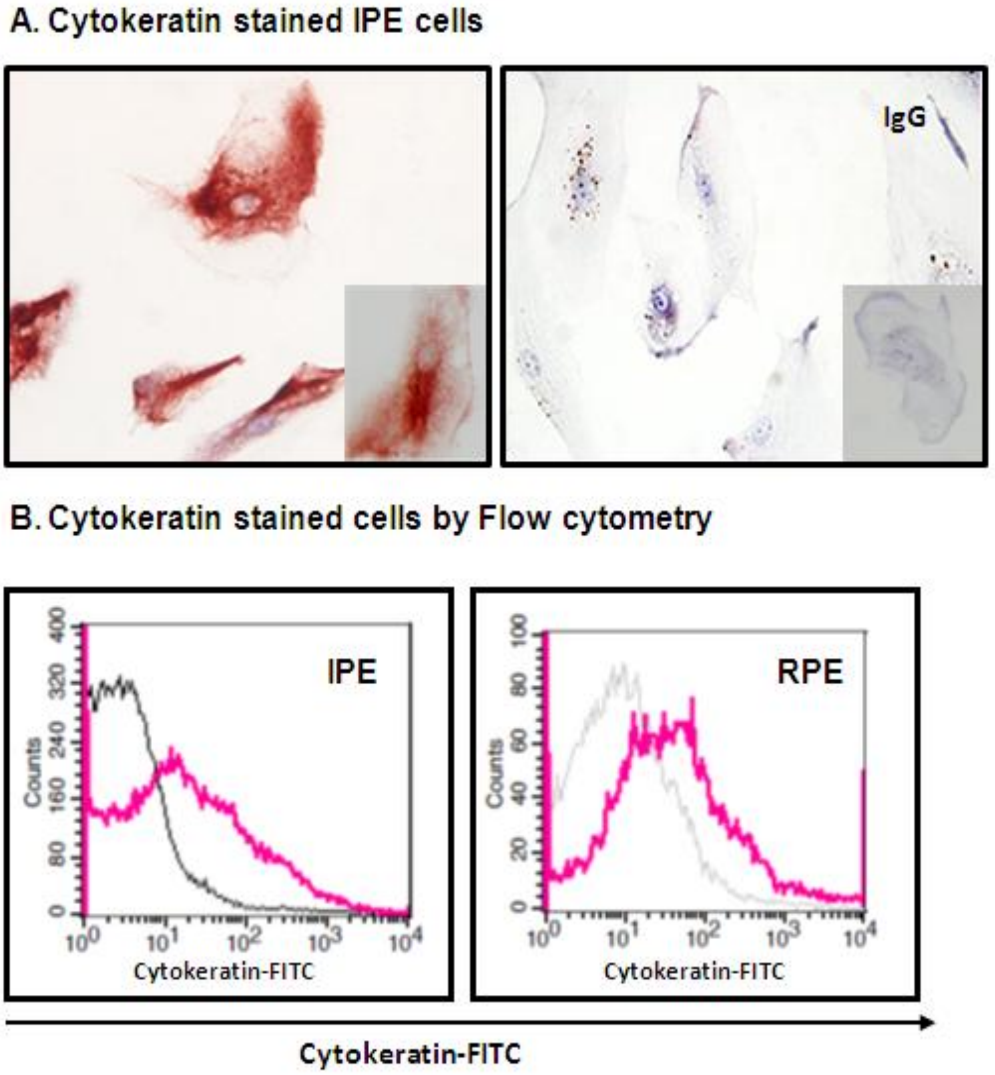

Supplement: Additional file 1 — Human IPE and RPE stained for cytokeratin. The primary IPE and RPE, cultured from iris and retina, of human donors, were probed with anti-pan cytokeratin antibody (1:200 dilution; A) and an isotype control antibody (mouse IgG1; 1: 200 dilution). Immunohistochemistry was performed to confirm the epithelial origin of primary IPE and RPE. A representative of cytokeratin stained IPE was shown in A and no staining was seen in the negative control (IgG1). The result was confirmed by flow cytometry (B). The peak representing cytokeratin stained cells (in pink) was shifted away from that of the isotype control antibody (in grey). [file 1476-9255-11-20-S1.pdf]
