# Supplementary material for: Role of toll-like receptors in human iris pigment epithelial cells and their response to pathogen-associated molecular patterns
Source: J Inflamm (Lond). 2014 Jul 16;11:20. doi: 10.1186/1476-9255-11-20 (PMC4118659; doi:10.1186/1476-9255-11-20)

File 2 Expression of TLR transcripts and proteins in human IPE and RPE. Human IPE (lane 1) and RPE (lane 2) from the same donor were cultured to confluence and expression of TLR1 to TLR10 genes and proteins was investigated by reverse transcription PCR (**A**) and Western blotting (**B**) using specific human TLR1 to 10 primers and antibodies, respectively. M= 100 bp DNA ladder (100, 200, 300, 400, 500, up to 1000 bp from bottom to top). TLR mRNA expression was measured by densitometry and normalised against GAPDH which served as a loading control. Normalised TLR mRNA expression levels are presented as mean ± SD (N=3) (**C**). Two-way ANOVA and Bonferroni's multiple comparison test were used to analyse the data, * $p< 0.05$, ** $p< 0.01$. Expression of TLR7 mRNA (A); TLR8 and 10 proteins (B) were not detected in both IPE and RPE. Results are representative of three experiments.

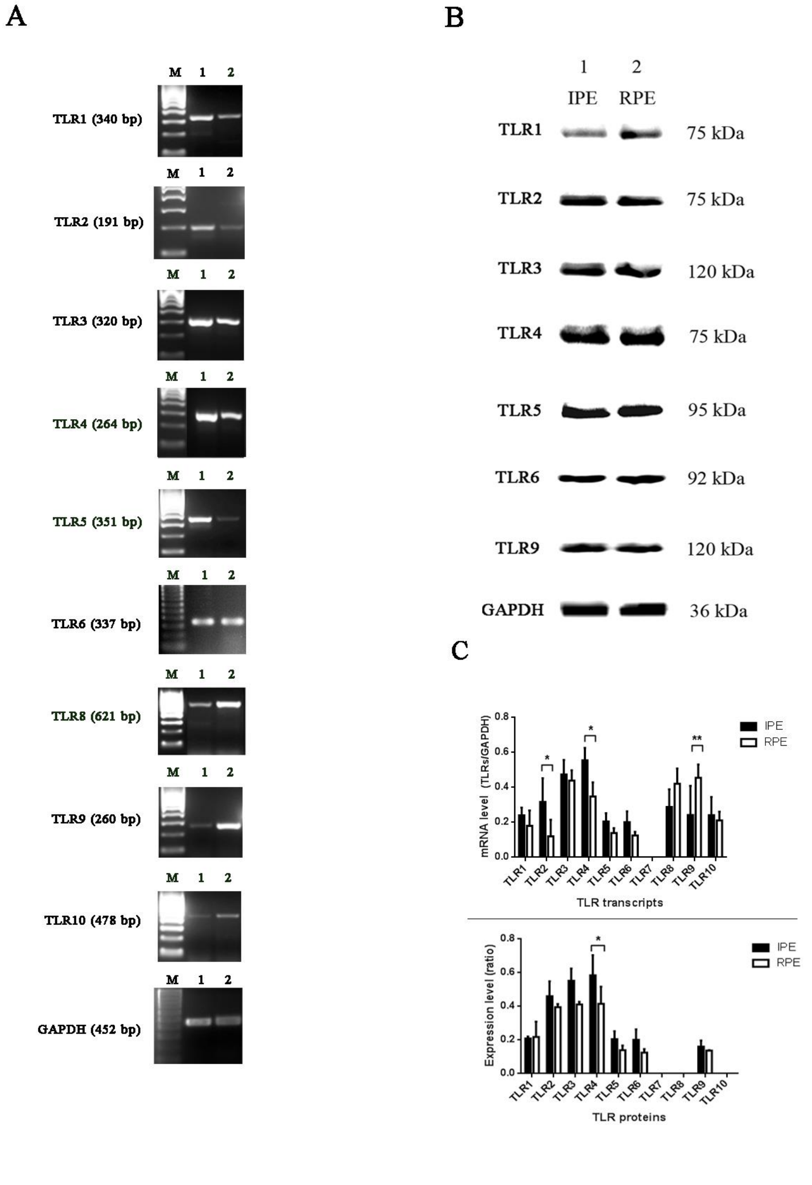

Supplement: Additional file 2 — Expression of TLR transcripts and proteins in human IPE and RPE. Human IPE (lane 1) and RPE (lane 2) from the same donor were cultured to confluence and expression of TLR1 to TLR10 genes and proteins was investigated by reverse transcription PCR (A) and Western blotting (B) using specific human TLR1 to TLR10 primers and antibodies, respectively. M= 100 bp DNA ladder (100, 200, 300, 400, 500, up to 1000 bp from bottom to top). TLR mRNA expression was measured by densitometry and normalised against GAPDH which served as a loading control. Normalised TLR mRNA expression levels are presented as mean ± SD (N=3) (C). Two-way ANOVA and Bonferroni’s multiple comparison test were used to analyse the data, *p< 0.05, **p< 0.01. Expression of TLR7 mRNA (A); TLR8 and TLR10 proteins (B) were not detected in both IPE and RPE. Results are representative of three experiments. [file 1476-9255-11-20-S2.pdf]
