# Supplementary material for: Role of toll-like receptors in human iris pigment epithelial cells and their response to pathogen-associated molecular patterns
Source: J Inflamm (Lond). 2014 Jul 16;11:20. doi: 10.1186/1476-9255-11-20 (PMC4118659; doi:10.1186/1476-9255-11-20)

File 3 Viability of IPE and RPE in the presence of OxPAPC, CI-095 and chloroquine.

Comment [KM1]: New results incorporated shown in C and F as a quality control for inhibition study

IPE and RPE were cultured in various concentrations of OxPAPC (**A** and **D**), CI-095 (**B** and **E**) and chloroquine (**C** and **F**) for 24 hours. The cells were subsequently detached from culture plates by trypsin, followed by assessment of viability using Trypan blue. Data represents mean ± SD (N=3). One-way ANOVA and Dunnett's post test was used to compare inhibitor-treated samples to controls. Both IPE and RPE remained ~90% viable in the presence of the high concentrations of TLR inhibitors. There was no difference in cell viability between control and inhibitor-treated cells.

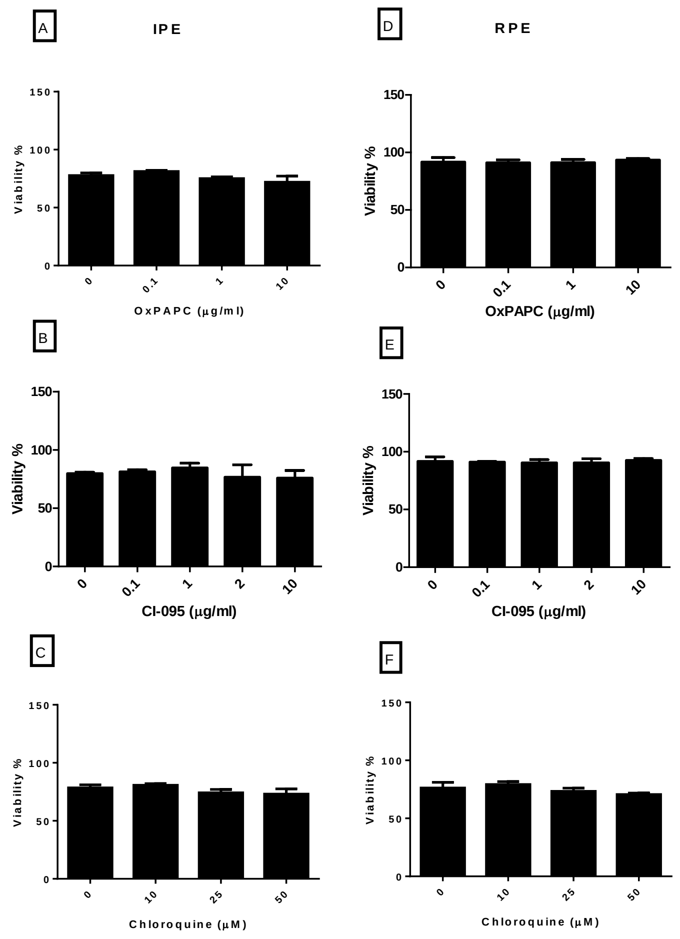

Supplement: Additional file 3 — Viability of IPE and RPE in the presence of OxPAPC, CI-095 and chloroquine. IPE and RPE were cultured in various concentrations of OxPAPC (A and D), CI-095 (B and E) and chloroquine (C and F) for 24 hours. The cells were subsequently detached from culture plates by trypsin, followed by assessment of viability using Trypan blue. Data represents mean ± SD (N=3). One-way ANOVA and Dunnett’s post test was used to compare inhibitor-treated samples to controls. Both IPE and RPE remained ~90% viable in the presence of the high concentrations of TLR inhibitors. There was no difference in cell viability between control and inhibitor-treated cells. [file 1476-9255-11-20-S3.pdf]
